# Antimicrobial Stewardship Programs in Chile: A Historical Overview

**DOI:** 10.3390/antibiotics15030247

**Published:** 2026-02-27

**Authors:** Mirta Acuña, Tania Herrera, Dona Benadof, Daniela Pavez, Luis Bavestrello, Ruth Rosales

**Affiliations:** 1 Hospital Roberto del Rio, Santiago 8380457, Chile; donaben@gmail.com; 2 Departamento de Pediatría y Cirugía, Facultad de Medicina, Universidad de Chile, Santiago 8380456, Chile; pavezdanita@gmail.com; 3 Centro de Investigación Clínica y Avanzada, Facultad de Medicina, Universidad de Chile, Santiago 8380456, Chile; 4 Sociedad Chilena de Infectología, Santiago 7500000, Chile; lbavestrello@gmail.com (L.B.); rurosalesch@gmail.com (R.R.); 5 Ministry of Health, Santiago 8320000, Chile; tania.herrera@minsal.cl; 6 Hospital San Juan de Dios, Santiago 8350567, Chile; 7 Facultad de Medicina Clínica Alemana, Universidad del Desarrollo, Santiago 7550000, Chile; 8 Clínica Reñaca, Viña del Mar 2540364, Chile; 9 Hospital Barros Luco Trudeau, Santiago 8900091, Chile; 10 Programa de Infectología, Universidad de Santiago de Chile, Santiago 9170022, Chile

**Keywords:** antimicrobial stewardship program, Chile, history, health policies

## Abstract

Antimicrobial Stewardship Programs (ASPs) are a cornerstone strategy to mitigate the global threat of antimicrobial resistance (AMR), primarily driven by inappropriate antimicrobial use. ASPs aim to optimize antimicrobial therapy by ensuring appropriate indication, agent selection, dosing, route of administration, and duration of treatment. Through these interventions, ASPs improve clinical outcomes, reduce adverse drug events, decrease selective pressure for resistant organisms, and contribute to healthcare cost containment. Effective implementation requires a multidisciplinary approach involving physicians, pharmacists, microbiologists, nurses, and information technology specialists, and must be tailored to local epidemiology and healthcare system capacity in accordance with World Health Organization (WHO) recommendations. This narrative review describes the development and evolution of the national antimicrobial stewardship policy in Chile, based on a review of publications indexed in SciELO, official documents from the Ministry of Health (MINSAL), and relevant national legislation. In Chile, antimicrobial stewardship initiatives began in the late 1990s with regulatory measures mandating prescription-only dispensing of antimicrobials and the introduction of national technical standards for rational antimicrobial use. After that, Chile adopted a comprehensive One Health approach and implemented national AMR action plans aligned with WHO strategies. Substantial progress has been achieved across hospital, primary care, veterinary, and aquaculture settings, including expanded ASP coverage, strengthened regulatory frameworks, national surveillance systems for antimicrobial consumption and resistance, and incorporation of stewardship indicators into institutional performance metrics. Despite these advances, challenges related to workforce capacity, technological infrastructure, and long-term monitoring persist and must be addressed to further consolidate national ASP implementation.

## 1. Introduction

ASPs are one of the main strategies developed to address the growing threat of AMR. The misuse of antimicrobials has contributed to the increase in this threat to public health [1].

These programs clearly improve healthcare through organized and systematic interventions implemented in healthcare institutions. ASPs aim to ensure the appropriate use of antimicrobials in terms of indication, drug selection, dosage, route of administration, and duration of treatment. The main objectives are to optimize clinical outcomes in patients with infections, minimize adverse effects related to antimicrobial use, reduce the selective pressure that favors the emergence of resistant microorganisms, and indirectly contribute to reducing healthcare costs [2,3,4].

Implementing an ASP requires a multidisciplinary approach, integrating various healthcare professionals such as physicians, microbiologists, pharmacists, nurses, and Information Technology Specialist, among others, while also adapting to the epidemiological context and the specific circumstances of each healthcare facility. Multiple international guidelines—including recommendations from the WHO, the Centers for Disease Control and Prevention, and the Infectious Diseases Society of America—have established guidelines for the design, implementation, and evaluation of these programs [4,5,6,7,8].

In Chile and other Latin American countries, ASPs are especially important given the high burden of healthcare-associated infections and the circulation of highly resistant pathogens [9,10]. Evaluating, strengthening, and implementing these initiatives is crucial for improving the quality of healthcare and optimizing the use of limited therapeutic tools in the context of multidrug-resistant bacteria. The aim of this narrative review was described the development and evolution of the national antimicrobial stewardship policy in Chile.

## 2. Results

### 2.1. Community Level

In Chile, concern about the appropriate use of antimicrobials dates back several decades. In the area of community consumption, the Antimicrobial Committee of the Chilean Society of Infectious Diseases (SOCHINF) promoted a study in 1997, led by Dr. Luis Bavestrello and Pharmacist Ángela Cabello, prior to the mandatory prescription to antimicrobials, which revealed a progressive increase in antimicrobial sales in private pharmacies between 1988 and 1997. This increase did not correlate with significant changes in the epidemiological profile of infectious diseases in the country, suggesting a gap between the optimal and actual use of these drugs [11].

These findings led to a coordinated effort involving the MINSAL and the SOCHINF, with the participation of other related scientific societies, the Public Health Institute, civil society consumer organizations such as the National Consumer Service (SERNAC), professional associations representing physicians and pharmacists, and members of the health committees of the Chamber of Deputies. The primary objective of this collaboration was to raise public awareness and promote initiatives aimed at strengthening state structures to ensure the rational use of antimicrobials nationwide.

**Figure 1:**
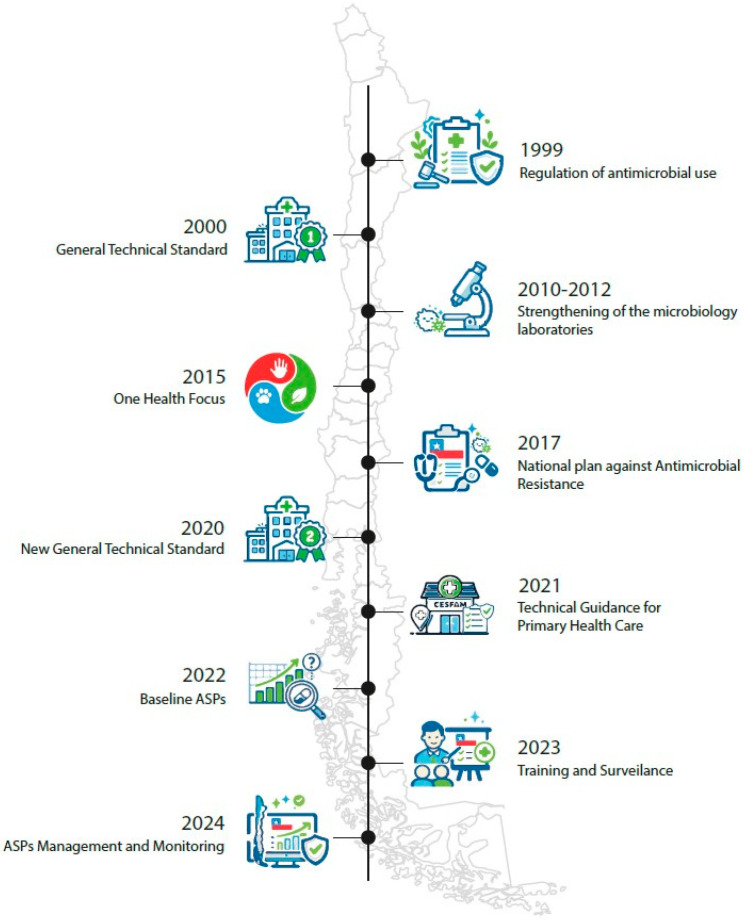
Development and strengthening of ASPs in Chile.

Following the presentation of this issue in multiple forums, including the National Congress, SERNAC, MINSAL, and a press conference held at the Medical Association, Ordinary Decree No. 4C/5051 (https://bcn.cl/3mjoa, accessed on 25 October 2025) was enacted in 1999, establishing mandatory prescription requirements for the dispensing of antimicrobials. This regulatory measure represented a major milestone in Chile, significantly transforming community-level antimicrobial use (Figure 1).

This regulation was pioneering in Latin America and in order to complement it with the appropriate use of antimicrobials in the hospital setting, it was followed in January 2000 by Exempt Resolution No. 2170 of the MINSAL, which approves the general technical standard No. 43 on “Rationalization of the use of antimicrobials in clinical care” [12] (Figure 1).

The analysis comparing the consumption density of antimicrobials between equivalent periods of the year prior to and after the promulgation of the Resolution demonstrated a significant decrease in the outpatient sale of antimicrobials [11], this confirmed the correct direction of the structural measures adopted at the national level to optimize antimicrobial use and appeared to be a fairly decisive measure. However, Bavestrello and Cabello in 2011 subsequently conducted a new study of antimicrobial consumption density in outpatient settings and surprisingly observed that after the third year following the enactment of the decree-law mandating a prescription for the purchase of antimicrobials, consumption began to increase again, returning within five years to levels similar to those of 1998 [13]. Analysis of the profile of this increase, concentrated primarily in prescription antimicrobials such as new macrolides and fluoroquinolones, revealed that self-medication was not the only problem in the inappropriate use of antimicrobials and that prescribing professionals, mainly physicians, were, to some extent, issuing antimicrobial prescriptions without the necessary clinical justification. This opened a new focus in the search for optimized antimicrobial use: regulation and education for both prescribers and the public.

In response, strategies are being developed to strengthen ASPs in primary health care; this is how, in 2021 a second version of the National Plan against Antimicrobial Resistance Chile 2021–2025 was published [14], this document highlights the existence and progress of ASP and establishes their strengthening within the community as a strategy for regulating and monitoring the use of antimicrobials in human health, companion and livestock, as well as in horticulture. It also emphasizes the need to promote research on AMR. Progress has also been made in community antimicrobial use through regulatory efforts in primary healthcare and strategies to control sales in unauthorized settings such as open markets, social media, and internet. In the same year, the Technical Guidelines for the Use of Antibiotics in Community-Acquired Infections Managed in Outpatient Settings were published [15], contributing to standardized management of common infections and serving as a training resource for prescribers (Figure 1). The following year, the MINSAL published the Technical Guidelines for the Implementation of ASP in Primary Care, a document approved by Exempt Resolution 199. This document urges primary care teams to create local ASP teams and develop work plans at that level to optimize antimicrobial use, while also promoting the establishment of an ASP team at the Health Service level to monitor the operation of local ASPs within their network [16].

### 2.2. The One Health

In 2015, the WHO recognized AMR as one of the top 10 global public health threats and launched the Global Action Plan on Antimicrobial Resistance [17]. This plan, developed in collaboration with the Food and Agriculture Organization of the United Nations and the World Organization for Animal Health, proposes a comprehensive approach based on “One Health,” addressing AMR in human, animal, and plant health, as well as the environment. Its objective is to ensure the treatment and prevention of infectious diseases with quality medicines, while promoting awareness and surveillance to reduce the spread of resistance. The plan urges all Member States, including Chile, to develop and implement national plans to address AMR in their own contexts (Figure 1).

In 2017, Chile launched its first National Plan against AMR with the participation of the Ministries of Health and Agriculture and the National Fisheries and Aquaculture Service. This plan is structured around five main strategic lines in accordance with the Global Action Plan. It includes the regulation and monitoring of antimicrobial consumption, giving impetus to the fight against AMR and, along with this, strengthening ASPs [18] (Figure 1).

Chile has adopted the One Health approach as a fundamental strategy for addressing AMR. One Health recognizes that human health, animal health, and environmental health are interconnected, and that AMR is a problem that cannot be addressed by a single sector. This approach has been key to the implementation of ASPs in the country.

In recent years, Chile has made significant progress in regulating antimicrobial use in animal production, especially in livestock and aquaculture, among others.

- Livestock: The Agricultural and Livestock Service (SAG) is the authority responsible for regulating the use of veterinary medicines in livestock farming. Regulations have been established that restrict the use of certain antimicrobials, including the prohibition of those considered critically important for human health by the WHO, such as some third- and fourth-generation cephalosporins and quinolones. Through its regulatory framework and responsible use programs, it actively promotes the reduction in antimicrobial use, discouraging widespread and unjustified prophylactic antimicrobial use. The focus is on therapeutic and metaphylactic use based on veterinary diagnosis, limiting administration to healthy animals only to exceptional and highly justified circumstances, and always under veterinary prescription and with the obligation to declare such use. The primary objective is to preserve the efficacy of antimicrobials for human and animal health [19].
- Electronic Prescribing: A key measure is the creation of the Electronic Prescribing System for Veterinary Antimicrobials, which aims to monitor and control the use of these products, making it mandatory for veterinarians to declare usage and biomass [20].
- Aquaculture: In Chile, the National Fisheries and Aquaculture Service (SERNAPESCA) and the Undersecretariat of Fisheries and Aquaculture (SUBPESCA) are the main agencies responsible for regulating the use of antimicrobials in the aquaculture sector, with a special focus on the salmon farming industry. The regulations aim to ensure animal welfare and control the emergence of AMR. There is a “General Health Program for the Use of Antimicrobials in Salmon Farming and Other Farmed Fish,” which establishes guidelines for the use of these drugs. This program prohibits the use of antimicrobials for fattening, growth, or preventative purposes, with the exception of metaphylaxis when there is a high risk of disease spread [21]. There is also a Program for the Optimization of Antimicrobial Use [22], this program consists of a voluntary certification awarded by SERNAPESCA to salmon farms that optimize antimicrobial use within established limits or eliminate their use entirely throughout the production cycle. Production cycles certified under PROA-Salmon have demonstrated significantly lower antimicrobial consumption than the industry average.
- Small animals: According to data from the World Organization for Animal Health, small animals, such as cats and dogs, consume almost 30% of the antimicrobials prescribed for animals. For this reason, in 2019, the first intersectoral working group was established to address bacterial resistance in small animals, with participants from scientific and technical organizations related to small animal health and infectious diseases (FAVET, SOCHINF, ISP, SAG, COLMEVET), and veterinarians from various universities. This work resulted in the 2021 publication of the Manual of Good Practices in the Use of Antimicrobials in Small Animals [23,24].

### 2.3. Hospital Level

Regarding human health and the hospital approach, there have been some studies in Latin America, such as that of Cabrera et al. in 2012, that demonstrate a decrease in antimicrobial consumption once an ASP is implemented [25]. Furthermore, another study demonstrated a wide dispersion in the consumption of intravenous antimicrobials among 29 hospitals in Chile, with diverse strategies and structures for optimizing and controlling the use of antimicrobials [26]. Aware of the existence of these and other scientific evidence, since 2017 the MINSAL has been working with the SOCHINF on the appropriate use of antimicrobials, evaluating current regulations and legislation. They observed that National Standard No. 43 was only being followed in a few healthcare facilities, likely because it did not include monitoring indicators or a follow-up plan from the central level, leaving its implementation dependent on the local enthusiasm of healthcare teams. Therefore, in subsequent years, work began on updating this standard, targeting hospitals across the country, incorporating updates from international evidence, the AWaRe classification, and indicators for ongoing monitoring. At the end of 2019 the text was practically ready, but we faced the global COVID-19 pandemic, which delayed its promulgation until 29 December 2020, with Exempt Resolution No. 1146 approving “General Technical Standard No. 210 for the Rationalization of the Use of Antimicrobials in Clinical Care” [27] (Figure 1).

The resolution established a period of six months to one year for installing the ASPs teams and beginning to implement the regulations in public and private hospitals of high and medium complexity, a deadline that was postponed due to the development of the pandemic, which exceeded hospitals capacity in the following years.

In 2022, a report was published on the baseline status of the ASPs in high and medium complexity hospitals [28] this report presents the results of the first survey designed and implemented by an external research team in collaboration with the MINSAL, serving as a baseline for verifying progress in this area. This survey was answered by 77 hospitals (56% of all high and medium complexity hospitals). It highlights that 83% (64) of the surveyed hospitals already had ASPs teams within their institutions, but only 66% (42/64) had a formal resolution issued by their institution’s management validating them, and 53% (41) had a written program outlining the activities to be carried out (Figure 1).

In 2024, further progress was made in establishing structures that support the development of ASPs, including their incorporation into the Management Commitments (COMGES). These mandatory commitments are agreements established between MINSAL and the country’s Health Services to evaluate the latter’s management, with the aim of improving their quality and efficiency. Its inclusion corresponded to COMGES 205 No. 16, “Quality and Safety in Care Plan”, which committed to the establishment of ASP teams within Health Services through approved and implemented work plans covering Primary Health Care and medium- and high-complexity hospitals. This measure strengthens institutional support [29]. These ASP indicators, which were included for the first time in the COMGES, were evaluated for compliance by each Health Service in the country, and their results are reflected in the “Antimicrobial Stewardship Program. COMGES Report 2024” [30] (Figure 1).

The results of a recent survey of 26 public hospitals in our country were published, highlighting that 92.3% (24/26) of them have an ASP team, with 95.8% (23/24) of these teams formally established. The majority include physicians (91.7%; 22/24) and a pharmacist (83.3%; 20/24). As was expected, antimicrobial consumption was higher in Intensive Care Units than in Internal Medicine Units. Among its conclusions, the survey emphasizes the need to review the staffing levels of healthcare professionals and incorporate technological tools, such as automated monitoring and process standardization [31].

In 2025, a Surveillance Report on Antimicrobial Consumption in Chile was published, analyzing data on the consumption of systemic antibacterials between 2022 and 2024 in public health institutions and private pharmacies, excluding private health institutions. This report shows that beta-lactams are the most consumed group of antibiotics in both sectors, and that 55.4 to 51.4% of antibiotics consumed belong to the Access group, which is below the goals proposed by the WHO (>70% of antimicrobial consumption should belong to the Access group) [32,33].

The Collaborative Group on Bacterial Resistance of the SOCHINF has also played an important role in monitoring antimicrobial resistance and use, promoting strategies for collecting information on the incidence of intensively resistant microorganisms and the density of antimicrobial consumption for more than a decade [34].

### 2.4. Training

Within the framework of the regional project “Working Together Against Antimicrobial Resistance,” a collaborative alliance was established in November 2021 with the Pan American Health Organization to strengthen the capacities of ASPs in hospitals. This led to multiple meetings to share best practices and provide training for conducting a point prevalence survey of antimicrobial use. The survey was finally carried out in 2023 with the voluntary participation of 13 hospitals in Chile and 54 hospitals in four other Latin American countries. Chile reported the lowest prevalence of antimicrobial use, at 39.0%, compared to the regional average of 47.9% [35]. Also in 2023, the ASP Implementation Course was launched, a 20-h online self-training course with content related to existing regulatory documents, which is currently hosted on the PAHO Virtual Campus, in order to facilitate access to training for the entire health team related to ASP [36] (Figure 1).

Furthermore, the SOCHINF Antimicrobial Committee has taken on the role of providing training on ASPs, including this topic in the most recent annual Antimicrobial Stewardship Courses, traditionally held every August in Chile. Additionally, SOCHINF has addressed ASPs in various modules at recent national congresses specializing in the field. On 28 November 2023, the SOCHINF board authorized the change in name of this Committee to the “Antimicrobial and ASP Advisory Committee,” thus highlighting and supporting this program.

In addition, recognizing the relevance of the topic and the need for training for teams at national level, a group from the Antimicrobial Advisory Committee and ASP, with the support of the industry, organized and held the very first ASP Summit Congress in 2023, a free training and experience exchange event. This was a free training and experience exchange event, which was a great success, with a large participation of teams from all Latin America regions, and with its second version in September 2025, with more than 300 attendees in person and more than 400 via streaming.

Advancing the consolidation of intersectoral work through Exempt Resolution 1045 of 24 July 2024, the MINSAL Advisory Group for ASP was created [37], which recognizes the collaborative work that has been developed with the teams of the healthcare network and the SOCHINF and also allows it to be strengthened for the coming years.

### 2.5. Pharmacist

The role of pharmaceutical Services in the ASP has been relevant. The WHO has highlighted community pharmacists as among the professionals best positioned to promote the appropriate use of antibiotics in the population, in coordination with scientific societies and health teams, and emphasizes that they have the necessary training and operate within the appropriate legal framework [38].

In Chile, since the incorporation of PROA programs in primary care in 2022, the necessary structure for pharmacist participation in the public sphere was established, marking a milestone that, to date, continues to develop [14].

On the other hand, in the private sector, its implementation remains a challenge and requires defining mechanisms that facilitate pharmacists in carrying out pharmaceutical education and care activities [39], considering the high participation of the outpatient setting in the total consumption of antimicrobials and the goal of maintaining ≥70% of consumption in drugs from the Access group according to the AWaRe classification [33,38].

In the hospital setting, there is a promising and relevant development of strategies for optimizing antimicrobials and pharmaceutical services, in accordance with publications that demonstrate the benefits of their incorporation into the appropriate use of antimicrobials [28,40].

### 2.6. Laboratory

The role of the Microbiology Laboratory is a fundamental pillar of ASPs, which provides support in timely microbiological diagnosis, guidance on the use of diagnostic tests, inflammatory response markers, selection of susceptibility testing panels, local susceptibility surveillance reports, collaboration in the interpretation of results, and education, among other things [41,42,43]. To this end, hospital microbiology laboratories have developed various strategies since the early 2000s to support the principles of ASPs.

One of the first and most significant contributions is the cumulative susceptibility study of clinically important pathogens, developed according to CLSI recommendations in document M39 [44]. This report is fundamental, as it forms the basis for defining empirical therapy regimens tailored to the local epidemiological reality, allowing for a more rational and effective selection of antimicrobials. This directly impacts the reduction in bacterial resistance, the optimization of clinical outcomes, and patient safety. Recognizing this importance, the Microbiology Committee of the SOCHINF published an executive summary in Spanish in 2010 with practical guidelines to facilitate the development and implementation of this document’s content in clinical laboratories in Chile [45].

On the other hand, the Chilean National Accreditation System, within the scope of “Access, Timeliness and Continuity of Care”, requires clinical laboratories of Institutional Providers of Outpatient and Inpatient Care to incorporate procedures to ensure the timely notification of risk situations detected through laboratory tests. Since its regulation, according to Decree No. 15 of 2007, this has allowed the implementation of notification of Gram stain results from positive blood cultures and sterile fluids to treating professionals, notifying them as a “critical value”. This is a mandatory characteristic of the process that allows for adjustment and optimization of antimicrobial use [46].

The evolution of bacterial resistance has posed significant challenges to bacterial identification and susceptibility studies, hindering the accurate testing of antimicrobials, interpretation, and the study of resistance mechanisms. Therefore, since approximately 2010, laboratories across the country have progressively optimized their microbiological diagnostic capabilities in bacterial identification and the incorporation of automated susceptibility testing, which allows for the determination of minimum inhibitory concentrations. Regarding the optimization of bacterial identification, mass spectrometry began to be incorporated in 2012, contributing to a reduction in bacterial identification time. The incorporation of molecular biology as a diagnostic tool has also contributed to reducing identification times and detection of resistance mechanism, along with an improvement in diagnostic sensitivity, which, in turn, has evolved in speed from real-time polymerase chain reaction to point-of-care testing. These strategies have contributed to optimizing antimicrobial use, helping to select the most appropriate antimicrobial for the patient (Figure 1).

Finally, it has been important for ASPs not only that laboratories optimize diagnostic technology and the speed of results, but also the administration and management of data, which has involved access to the statistics needed periodically for developing ASP indicators and surveillance [47].

## 3. Discussion

The Chilean experience provides a relevant narrative example of the progressive evolution of antimicrobial stewardship from isolated regulatory actions toward a comprehensive, multisectoral, and system-integrated strategy to address AMR. Early national data revealing a sustained increase in community antimicrobial consumption, unrelated to epidemiological trends, served as a critical trigger for policy action and highlighted the disconnect between evidence-based prescribing and real-world practice. The introduction of mandatory prescription-only antimicrobial dispensing in 1999 represented a landmark regulatory intervention in Latin America and demonstrated that structural measures can rapidly reduce inappropriate access and consumption.

Chile’s later alignment with the WHO Global Action Plan and the formal adoption of the One Health framework marked a pivotal expansion of stewardship beyond human healthcare [17,18]. Regulatory advances in livestock production, aquaculture, and companion animal medicine—particularly restrictions on critically important antimicrobials and the implementation of electronic veterinary prescribing systems—illustrate the value of coordinated intersectoral governance in reducing antimicrobial selective pressure across ecosystems. These strategies and actions, framed within the National Plan Against Antimicrobial Resistance, have also had some impact on the population. Among consumers in Chile, particularly those under 45, there is some awareness that the use of antimicrobials in food-producing animals should be regulated [48].

Within hospitals, the delayed but eventual modernization of stewardship regulations, incorporating international evidence, the AWaRe classification, and measurable performance indicators, enabled more consistent ASP implementation nationwide. National surveys demonstrate high ASP coverage and formalization; nevertheless, persistent challenges related to workforce allocation, protected time, and technological infrastructure remain evident. The consistently higher antimicrobial consumption in critical care settings further emphasizes the need for targeted, unit-specific stewardship interventions.

The implementation of ASPs supported by public health policies in Chile has proven to be a feasible and effective strategy; however, development faces relevant barriers, including limitations in specialized human resources, heterogeneity in hospital microbiological capacity, and organizational resistance to change. Key facilitators include the centralized health model in Chile, with network management, the presence of standardized national guidelines, the establishment of formal multidisciplinary stewardship teams, and the integration of ASPs into national antimicrobial resistance containment strategies, which have enabled reductions in broad-spectrum antimicrobial consumption and associated healthcare costs. The transferability of this model to other health systems appears plausible, particularly in settings with centralized governance structures and basic microbiological surveillance capacity; nevertheless, its adoption may be constrained by differences in healthcare financing, availability of trained specialists, and local epidemiological contexts.

In other countries such as the United States, its implementation has been consolidated through the CDC’s “Core Elements” [49] and regulatory requirements linked to hospital accreditation and federal funding, which fosters widespread adoption. In the European Union, the policy is structured through the One Health approach, with supranational plans, quantifiable targets for 2030, and comparable surveillance systems across countries [50]. In Latin America, development is based on technical support from the Pan American Health Organization (PAHO), regional surveillance networks, and heterogeneous national plans, where some countries have established specific ministerial regulations for Antimicrobial Resistance Prevention (ARP) [5]. Taken together, these experiences demonstrate different public policy models for addressing antimicrobial resistance, combining regulation, epidemiological surveillance, and strengthening clinical practice.

## 4. Materials and Methods

This narrative review was conducted based on a search of publications indexed in SciELO, and PubMed. Additionally, in official documents from the Ministry of Health (MINSAL), and relevant national legislation related to Antimicrobial Stewardship Programs in the National Congress Library. The time frame was between 18 October 2025 and 17 February 2026, the language was restricted to Spanish and English.

The MeSH words included were antimicrobial stewardship programs, Chile, MINSAL, PROA, One Health and antimicrobial resistance.

## 5. Conclusions

Chile has achieved sustained progress in implementing ASPs in community and hospital settings, in both the public and private sectors. This development has taken almost 30 years of multidisciplinary and intersectoral work and has been supported by public policies such as the early development of regulatory measures, national technical standards, and the integration of ASPs into public and private hospitals. The incorporation of ASP indicators into national management frameworks has strengthened the program’s sustainability. The adoption of the One Health approach has enhanced the achievements in antimicrobial stewardship in human, animal, and environmental health. The Chilean experience offers transferable lessons for middle-income countries seeking to move from regulatory compliance to resilient, data-driven, and multidisciplinary antimicrobial stewardship systems. Despite these advances, challenges remain in achieving consistent implementation, ongoing staff training, and systematic dissemination of results.

## Data Availability

No new data were created; the data used in this manuscript are available in the bibliographic citations.

## Abbreviations

The following abbreviations are used in this manuscript:

ASP	Antimicrobial Stewardship Program
AMR	Antimicrobial resistance
WHO	World Health Organization
MINSAL	Ministry of Health
SOCHINF	Chilean Society of Infectious Diseases
SERNAC	National Consumer Service
SAG	Agricultural and Livestock Service
SERNAPESCA	National Fisheries and Aquaculture Service
SUBPESCA	Undersecretariat of Fisheries and Aquaculture
COMGES	Management Commitments
PAHO	Pan American Health Organization
